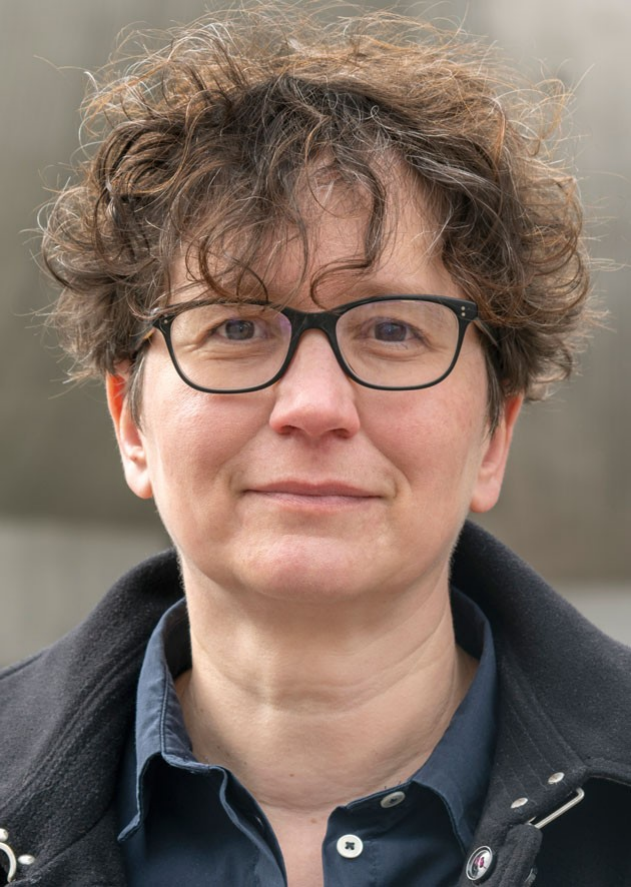# Time‐Resolved In Situ Monitoring of Mechanochemical Reactions

**DOI:** 10.1002/anie.202117270

**Published:** 2022-04-05

**Authors:** Adam A. L. Michalchuk, Franziska Emmerling

**Affiliations:** ^1^ BAM Federal Institute for Materials Research and Testing Richard-Willstätter-Strasse11 12489 Berlin Germany; ^2^ Department of Chemistry Humboldt-Universität zu Berlin Brook-Taylor-Strasse 2 12489 Berlin Germany

**Keywords:** Kinetics, Mechanism, Mechanochemistry, XRD, in Situ Analysis

## Abstract

Mechanochemical transformations offer environmentally benign synthesis routes, whilst enhancing both the speed and selectivity of reactions. In this regard, mechanochemistry promises to transform the way in which chemistry is done in both academia and industry but is greatly hindered by a current lack of mechanistic understanding. The continued development and use of time‐resolved in situ (TRIS) approaches to monitor mechanochemical reactions provides a new dimension to elucidate these fascinating transformations. We here discuss recent trends in method development that have pushed the boundaries of mechanochemical research. New features of mechanochemical reactions obtained by TRIS techniques are subsequently discussed, which sheds light on how different TRIS approaches have been used. Emphasis is placed on the strength of combining complementary techniques. Finally, we outline our views on the potential of TRIS methods in mechanochemical research, towards establishing a new, environmentally benign paradigm in the chemical sciences.

## Graphical abstract

Time‐resolved in situ analysis opens the black box. Within the last few years, time‐resolved in situ (TRIS) analysis has revolutionized the field of mechanochemistry. This Minireview highlights the insights gained in mechanochemical transformations, thereby paving the way for future academic and industrial breakthroughs of mechanochemisty as a green and sustainable approach for designing new materials.

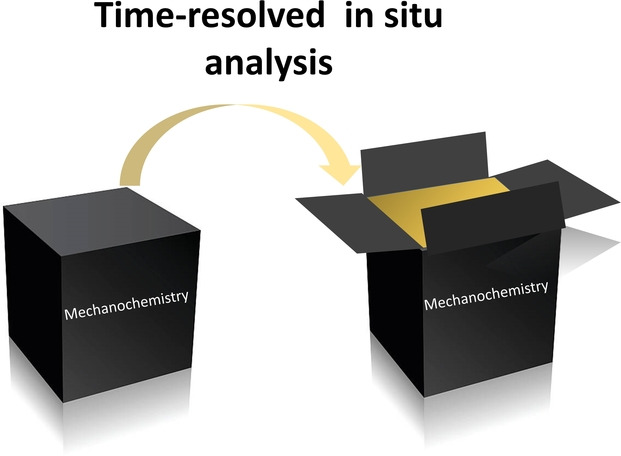

## 1 Paradigms of Studying Mechanochemical Reactions

Our ability to induce chemical and physical transformations using mechanical force pre‐dates historical records. In fact, the very first chemical reaction conducted by early humans—making fire—was done “mechanochemically”. Despite its immense history, the field of mechanochemistry remained largely a curiosity until the end of the 19th century and only expanded as a scientific discipline during the 20th century.^[1]^ The last two decades have seen mechanochemistry grow to become a popular environmentally benign alternative to solution‐based synthesis methods. For example, grinding and ball milling in the absence of solvent (neat grinding) or with a small amount of liquid (liquid‐assisted grinding, LAG) or polymers (polymer‐assisted grinding, POLAG) is highly efficient for preparing inorganic, metal‐organic, and organic compounds; detailed discussions can be found in recent reviews and chapters.[^2^, ^3^, ^4^] Moreover, mechanochemical methods can provide routes to products that are otherwise inaccessible by solution techniques. In recognition of the exceptional potential of this sustainable synthesis strategy, IUPAC has dubbed mechanochemistry as one of the “ten chemical innovations that will change our world”.^[5]^

**Figure 1:**
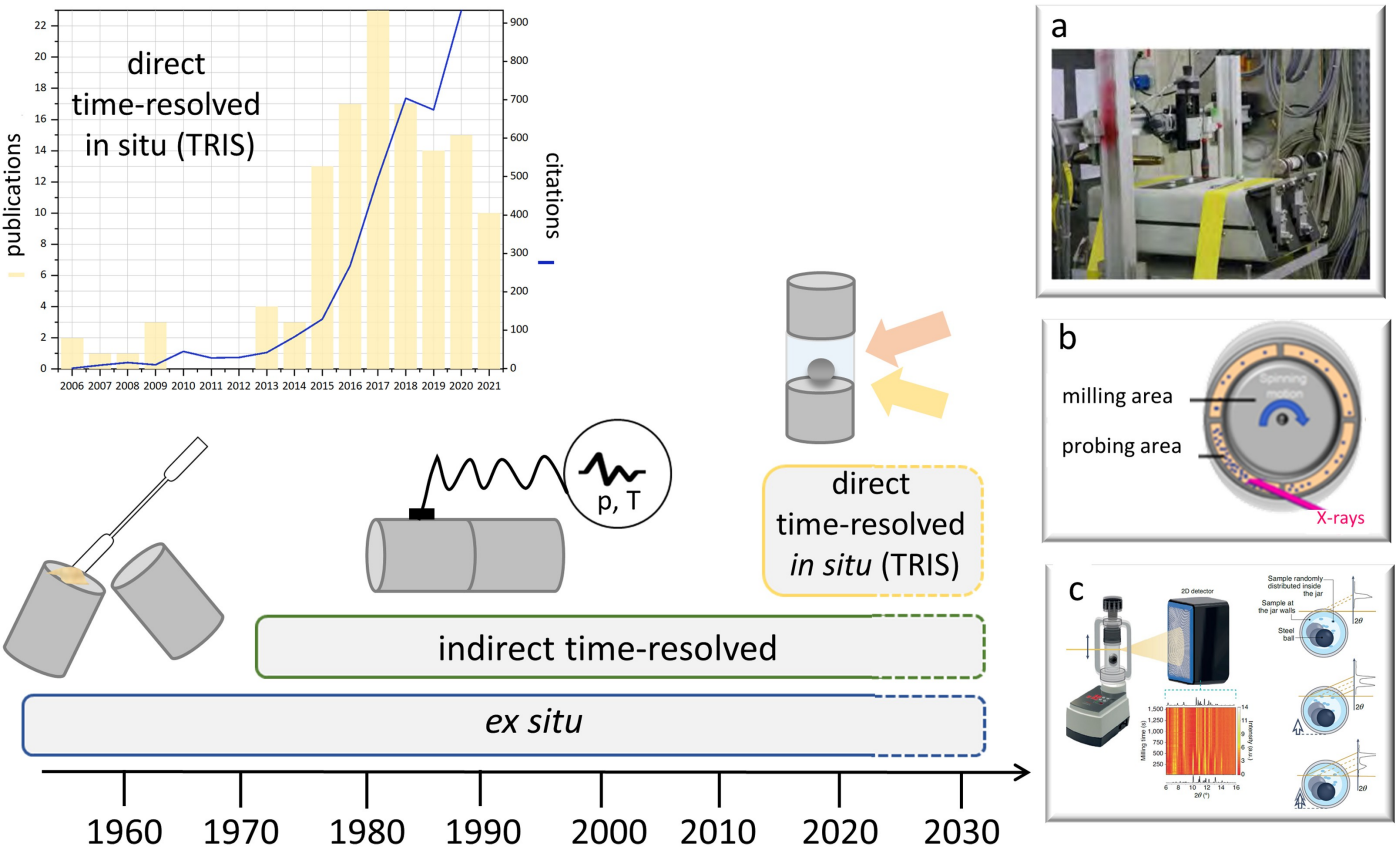
The application of ex situ and in situ methods over the years. Left inset: the number of papers in Web of science each year in which “in situ” and “mechanochemistry” are used in the title, abstract, or as keywords. a) First in situ XRD experiment including a commercial instrument adapted for use at the synchrotron;^[10]^ Copyright 2021 Nature Publishing Group. b) Customized milling container with a continuous “probing” ring for enhanced XRD data for neat grinding experiments;^[11]^ Copyright 2017 American Chemical Society. c) The recently optimized sample environment and measuring strategy for XRD data.^[12]^ Copyright 2021 Nature Publishing Group.

Although mechanochemistry is promising as an environmentally benign method, the lack of understanding of mechanochemical transformations poses significant restrictions on its use in academia and industry. The mechanistic understanding of mechanochemical reactions is sparse and has been acquired mostly by stepwise ex situ analysis (Figure 1). However, many transformations occur too quickly to be captured by ex situ analysis, may be unstable to atmospheric conditions, or may change when extracted from the milling apparatus. Only through time‐resolved in situ methods can the information be obtained that is required to realize the full potential of this transformative technology.

### 1.1 The Ex Situ Analysis Paradigm of Mechanochemistry

Most commonly, the product of a mechanochemical reaction is analyzed ex situ by removing a sample from the mechanochemical reactor. As such, there are effectively no restrictions on which analytical techniques can be used to characterize mechanochemically treated samples. Many aspects of mechanochemical transformations have been, therefore, studied ex situ, including detailed studies of how milling parameters (frequency, energy, additives) influence the outcome of a milling reaction,[^6^, ^7^] alongside investigations on the origins of mechanical reactivity (Figure 1).[^8^, ^9^]

Despite their widespread use, ex situ analyses are always accompanied by several caveats. Notably, one does not actually measure the state of the system under the reaction conditions.^[13]^ Instead, assumptions must be made to correlate the final state of the material to the mechanochemical conditions. Moreover, many mechanically driven transformations are known to continue even after mechanical treatment has stopped.[^14^, ^15^, ^16^, ^17^] Kinetic analyses—although successfully performed in many cases^[18]^—must inherently assume that post‐reaction analyses accurately reflect the state within the reactor. At the same time, difficult sampling from ex situ analysis greatly limits the resolution of data for high‐quality kinetic analysis.

Even very simple assumptions must be made when conducting ex situ analysis. For example, a mechanochemical transformation comprises an interplay between the structure of the powder (e.g. mixing and rheology) and the chemical processes themselves.^[19]^ As such, even the very act of removing material from the reactor can alter the course of the reaction.[^20^, ^21^] For this reason, methods to study mechanochemical transformations in situ (i.e. inside the reactor vessel) have been proposed. An early example is that by Ma et al., who studied the ball‐milling synthesis of the prototypical zeolitic imidazole framework (ZIF) ZIF‐8.^[22]^ Using a hand‐held Raman spectroscopy probe, the reaction rate was followed by stopping the mill and inserting the probe directly into the reaction jar, thereby avoiding disturbance of the powder material. Although these developments do bypass problems with sampling, the stop‐start methodology of the ex situ paradigm remains.

### 1.2 The Time‐Resolved In Situ (TRIS) Paradigm of Mechanochemistry

**Figure 2:**
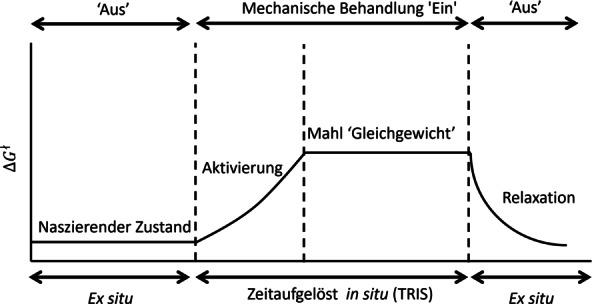
The influence of mechanical treatment on the energetics of solids. Mechanical treatment drives the solid away from equilibrium into an energetically “activated” state.^[24]^ Once mechanical treatment is stopped, this excess energy is allowed to relax, although the relaxed state may still be “activated” with respect to the nascent state.^[13]^

Mechanochemical reactions rely on a balance between the mechanical excitation of the material and the subsequent relaxation of this activated state.[^13^, ^23^] During continuous mechanical treatment (e.g. ball milling), periodic mechanical stresses generate a balance between excitation and both the relaxation and thermal annealing processes. This balance drives the system into an energetically activated “steady state” (or “mechanochemical equilibrium”) that remains in place as long as the mechanical treatment remains uninterrupted and unaltered (Figure 2).[^24^, ^25^] Characterizing the processes that lead to this steady state,[^12^, ^17^, ^26^] as well as its nature,[^7^, ^27^] hold the key to better understand the driving forces behind mechanically driven solid‐state transformations. These goals can only be achieved by following mechanochemical transformations through time‐resolved in situ (TRIS) methods.

Interest in following mechanochemical transformations in situ and in real time already began in the 1980s.^[24]^ Early efforts focused on assessing the conditions inside mechanochemical reactors, primarily during treatment by means of temperature and pressure. Thermal analysis was used, for example, to follow the formation and self‐propagating mechanism of ball‐milled powders.^[28]^ Similarly, changes in gas pressure allowed the progress of a reaction to be followed.^[29]^ Although insightful, these methods provided only indirect measures for processes occurring within mechanochemical reactors. Further mechanistic insight requires direct structural information.

Mechanochemical reactions are predominantly explored with solid materials. Correspondingly, efforts to access direct structural information during mechanochemical reactions began with the development of TRIS‐X‐ray diffraction in 2013.^[10]^ This was followed soon after by demonstrations of TRIS‐Raman spectroscopy, which quickly expanded to a range of complementary analytical technologies. Some early developments in TRIS monitoring of mechanochemistry were discussed in 2015,^[30]^ wherein the authors focused on the description of the first in situ experiments using synchrotron X‐ray powder diffraction and Raman spectroscopy. With significant advances in technologies since then, including the development of entirely new tools and methods, we here provide the field with an update on the strides made using TRIS techniques in mechanochemistry.

## 2 Methods for TRIS Monitoring of Mechanochemistry

**Figure 3:**
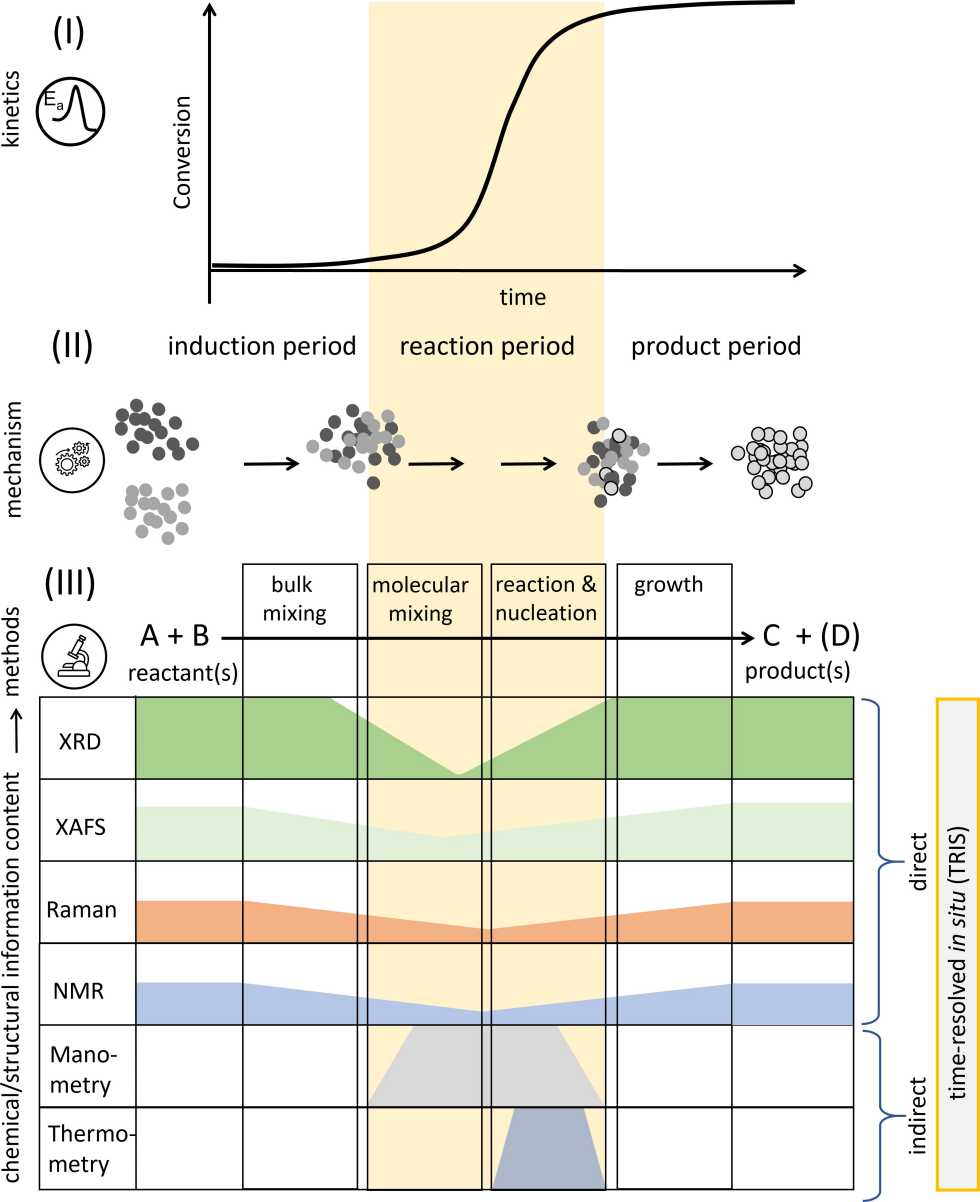
General evolution of mechanochemical transformations and the TRIS methods available to study them. I) A schematic generic kinetic profile for a mechanochemical transformation, comprising an induction period, reaction period, and product period. II) Schematic representation of the macroscopic mechanism describing a generic mechanochemical transformation, comprising bulk mixing, molecular mixing, reaction and nucleation, and growth phases. III) Existing TRIS methods to characterize mechanochemical transformations, with indications of their strength to characterize different reactivity regimes.

A mechanochemical transformation involving solid reagents follows a complex series of stages. 1) The solid materials must mix to generate contacts between different reagents. This stage involves both macroscopic mixing (i.e. movement of solid particles) and microscopic mixing through powder comminution and the formation of mobile intermediate phases such as amorphous phases, liquids, or gaseous products. 2) Once mixed at the atomic or molecular level, the physicochemical transformation can occur. 3) The product phase forms as a crystalline solid, starting first as a nucleus and growing into a nano‐ or microcrystalline phase. Although these stages are certainly an oversimplification of the process, they serve well to highlight the important aspects of TRIS monitoring in mechanochemistry. Importantly, these three stages give rise to the general features of the reaction profiles observed for mechanochemical transformations:[^12^, ^31^] 1) an induction period during which no transformation is observed, 2) a growth phase during which chemical and physical transformations occur, including chemical reactions, nucleation, and growth, and 3) a plateau, signifying the end of the physicochemical transformation (Figure 3).

Mechanochemical reaction profiles have been followed using various TRIS experimental methods, including manometry,[^32^, ^33^, ^34^] thermometry,[^35^, ^36^] X‐ray[^10^, ^12^, ^37^] and neutron^[38]^ diffraction, and vibrational,[^39^, ^40^, ^41^, ^42^] nuclear magnetic resonance,^[43]^ and X‐ray absorption^[44]^ spectroscopy. Whereas techniques based on manometric and thermometric measurements provide an indirect measure of reaction progress, the remaining techniques offer direct structural detail regarding mechanochemical transformations. Moreover, each technique has its own strength for probing different aspects of the transformation (Figure 3). We here provide only a brief overview of the main, currently available analytical techniques, and refer the readers to more in‐depth discussions in Ref. [45].

### 2.1 Methods for Direct Analysis

*X‐ray Powder Diffraction (XRD)*. First demonstrated in 2013,^[10]^ high‐intensity synchrotron X‐ray radiation can penetrate ball‐milling jars and be scattered by the powdered material within (Figure 1a). As a consequence of the broad path taken by the beam through the milling jar, scattering occurs from the sample at multiple positions and thus leads to artificial broadening (or even splitting) of Bragg reflections.^[12]^ Early efforts attempted to correct this splitting analytically.^[46]^ An alternative milling geometry was proposed, wherein the beam is focused through a small Kapton (polyimide) compartment on the outer circumference of the vessel (Figure 1b).^[11]^ With an exceptionally thin width, this construction has, to date, provided the highest quality X‐ray diffraction data, analogous to data collected from capillary measurements. However, an unhindered exchange of powder between this sampling compartment and the bulk reactor is required. The setup is, therefore, not amenable to studying mixtures that contain liquid (liquid‐assisted grinding), are hygroscopic or produce liquid,^[47]^ or may melt during the reaction. Inspired by the success of the approach, efforts to enhance the signal to background ratio were made by minimizing the beam path through the reactor vessel.^[48]^ By focusing the X‐ray beam across the shortest path, the ideal single‐point scattering can be approximated (Figure 1c). This approach has been recently extended to include innovations in data treatment strategies, with data collection strategies now offered for TRIS‐XRD that are competitive with ex situ analysis.^[12]^

Despite significant progress, many challenges remain for TRIS‐XRD. During a TRIS‐XRD experiment, the X‐ray beam is focused through a single (very small) portion of the milling jar. Correspondingly, only material that passes through the beam is measured, intrinsically assuming homogeneity in the reactivity of the sample. This assumption is not always appropriate, as has been shown by comparing in situ and ex situ phase analysis.^[20]^ Moreover, TRIS‐XRD methods are currently limited to reactors with relatively small vessel displacements and cannot yet be applied to reactors such as planetary ball mills and twin‐screw extruders, which display different mechanical activation types. Another challenge associated with TRIS‐XRD analysis is the need to use X‐ray‐transparent sample vessels, which are generally comprised of a polymeric material such as PMMA. In contrast to laboratory experiments which are typically performed in steel vessels, the polymeric material can alter the outcome of mechanochemical reactions.[^21^, ^49^, ^50^] Moreover, polymeric materials are too soft for mechanochemical treatment of hard inorganic phases, and alternative vessels capable of circumventing these problems are required. Progress in this direction has been reported recently by Rathmann et al.,^[51]^ who designed stainless‐steel milling jars with X‐ray‐transparent polymer windows, sufficiently robust to withstand the milling of hard inorganic solids during TRIS‐XRD analysis.

*Raman Spectroscopy*. As a laser‐based method, Raman spectroscopy is well‐suited for in situ investigations as the laser can easily penetrate an optically transparent sample vessel. In contrast to TRIS‐XRD, TRIS‐Raman spectroscopy is not restricted to the study of crystalline materials, thus making it an excellent complementary technique.^[52]^ By measuring the vibrational spectrum of a material, TRIS‐Raman spectroscopy provides a probe into the chemical structure (i.e. formation or breaking of new bonds) of the material, thereby making it well‐suited for studying chemical transformations[^53^, ^54^, ^55^] or the formation of multicomponent materials (e.g. cocrystals) through the generation of new, strong intermolecular interactions.^[56]^ However, its sensitivity to changes in the crystallographic structure of a material is much poorer than XRD. For this reason, TRIS‐Raman spectroscopy is not always suitable for studying solid‐state transformations, such as polymorphism, where no new covalent bonds are formed or broken. This challenge has been solved in part through developments in advanced data analyses, such as principal component analysis.[^52^, ^57^] For a detailed discussion on TRIS‐Raman spectroscopy, we refer readers to a recently published protocol.^[40]^

*NMR Spectroscopy*. A relatively new approach to monitor mechanochemical transformations is the use of time‐resolved solid‐state NMR (ssNMR). First reported by van Wüllen and co‐workers,^[58]^ the authors realized static ssNMR measurements during a prototypical mechanochemical reaction (formation of zinc phenyl phosphonate) by introducing a miniaturized ball mill in a self‐made ssNMR probe. With this setup, product formation was monitored over time and the influence of the number of milling balls and milling frequency was studied quantitatively. Leger et al.^[43]^ proposed another in situ approach for mechanochemical processes, through the introduction of magnetic resonance relaxation time correlation measurements to access the hydrogen environments of reactants and products. The authors illustrated the possibilities of the method by investigating benchmark metal‐organic framework (MOF) systems.

**Figure 4:**
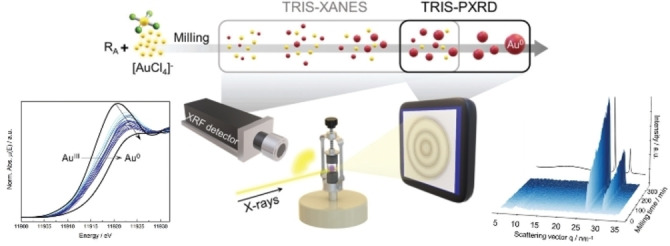
Tandem TRIS‐XANES and TRIS‐PXRD setup for the investigation of the bottom‐up synthesis of gold nanoparticles. Figure adapted from Ref. [44]with permission from the Royal Society of Chemistry.

*X‐ray Absorption Spectroscopy*. To complement synchrotron‐based X‐ray diffraction methods, de Oliveira et al.^[44]^ demonstrated that milling transformations could also be monitored by X‐ray absorption spectroscopy (XAS; Figure 4). By using X‐ray fluorescence, the X‐ray absorption near edge spectra (XANES) were measured at the Au L_III_‐edge during the bottom‐up mechanochemical synthesis (BUMS) of Au nanoparticles. In this way, the authors could track the mechanochemical redox reaction at the local atomic level, before crystalline phases became visible by TRIS‐XRD.

TRIS‐XAS promises great potential for mechanochemical investigation. This approach is still in its infancy, and many developments are still required. Owing to issues with sample inhomogeneity and absorption, it is currently only possible to collect TRIS‐XAS data through fluorescence. This greatly limits the temporal resolution (minutes) compared with TRIS‐XRD (seconds) and restricts measurements to the near‐edge region (XANES). Moreover, XAS is inherently plagued by the need to measure at the absorption edge of the material, thereby greatly reducing the intensity of the signal. This is particularly problematic for absorption edges of less than about 10 keV, where conventionally used milling jars (and air) also absorb strongly.

### 2.2 Methods for Indirect Analysis

*Manometry*. Manometric measurements, that is, the measurement of pressure changes over the course of the reaction, have been applied to mechanochemical research for many decades.[^29^, ^34^, ^59^, ^60^, ^61^] This method relies on the consumption or production of gas over the course of the mechanochemical transformation. The change in gas pressure is measured using a pressure sensor that is fitted into the reaction jar during milling. Although providing only indirect evidence for chemical and physical transformations within the sample jar, manometry has provided strong experimental support for elucidating mechanochemical mechanisms. In the case of ball milling Zn and S_8_, XRD was only able to show changes in the crystallographic structure. Only through the tandem use of manometric measurements could the authors conclude that one reactant phase (S_8_) volatilized during milling, thereby forming a reactive intermediate fluid phase to drive formation of the final product.^[62]^

*Thermometry*. Thermal measurements during mechanochemical experiments have been performed for many years. Data were obtained using thermocouples and sensors attached to the milling vessel[^56^, ^57^, ^58^, ^59^] or by measuring the temperature of the vessel and milling balls directly after termination of the experiment.[^64^, ^67^]

Mack and co‐workers presented an elegant experiment that correlated the rate of a Diels–Alder reaction in solution to the rate of the same reaction under mechanochemical conditions. By comparing reactions with different starting materials and calculating the activation energies for each reaction, they were able to assign a maximum energy output for the mill used between 95.4 and 111.7 kJ mol^−1^. Performing the reaction in solution at 90 °C for 16 h led to the same yield as a mechanochemical reaction in a stainless‐steel vessel with one milling ball in the same time frame. The authors concluded that the conditions within the mill are comparable to those of the same reaction performed in solution.^[63]^

Using a known mechanochemically induced polymorphic transformation of prototypical MOFs, Užarević et al. followed the composition and temperature changes by combining TRIS‐XRD and temperature measurements, finding again that changes in the temperature profile correlated with changes in the reaction mixture.^[68]^

## 3 What Has Been Learnt from TRIS Investigations?

**Figure 5:**
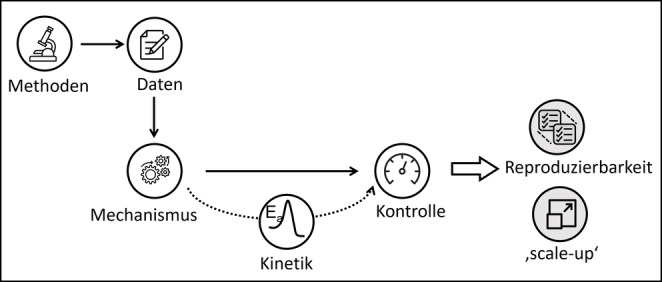
A degree of control is required to achieve reproducible and scalable mechanochemical processes. This can only be achieved through detailed understanding of the underlying mechanisms, which is attainable through analytical investigations.

An overarching aim in modern mechanochemical research is to obtain control over these environmentally benign reactions (Figure 5). In this way we hope to establish reproducible and scalable processes suitable for real‐world applications. At its core, gaining control requires a thorough understanding of the underlying mechanisms. These mechanisms can be studied directly or indirectly, for example, by mapping the reaction kinetics. Once a mechanism is understood, its dependence on the mechanochemical parameters can be subsequently studied to ultimately achieve some degree of control. In the following section we highlight how TRIS methods have been used to tackle each of these stages towards achieving control over mechanochemical reactions.

### 3.1 Mechanisms

When discussing mechanochemical reactions, the term “mechanism” can be taken to describe at least two aspects of the transformation: first are the “chemical” aspects, including the presence and reactivity of chemical species, and second are the “physical” aspects, such as the structure (state of mixing, rheology, etc.) of the powder material itself. TRIS has become irreplaceable for both avenues of investigation.

**Figure 6:**
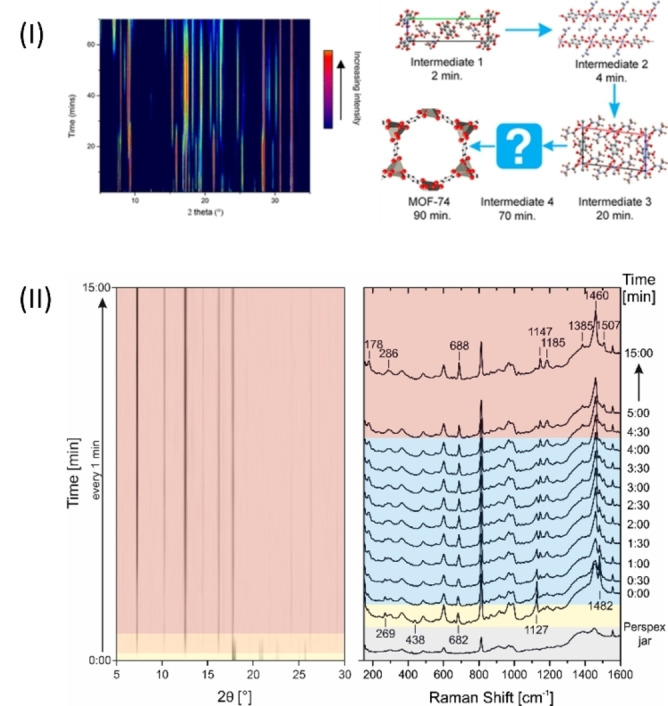
Applications of TRIS‐XRPD to follow ball‐milling syntheses. I) Mechanochemical formation of MOF‐74, characterized by TRIS‐XRPD. The time‐resolved diffractograms (left) show a complex interconversion of intermediates prior to formation of the final MOF‐74 phase. Figure modified from Ref. [69] with permission. Copyright 2021 American Chemical Society. II) Mechanochemical synthesis of ZIF‐8, as followed by TRIS‐XRD (left) and Raman spectroscopy (right). Yellow: reactants, orange: reactants and product ZIF‐ 8, red: product ZIF‐8, blue: product ZIF‐8 with not yet perfectly arranged 2‐methylimidazolate molecules within the crystal structure. Figures modified from Ref. [56] with permission. Copyright 2015, Wiley‐VCH.

*Chemical Aspects*. In principle, reactions that follow simple profiles, for example, A+B→C , can be easily followed by ex situ analyses. However, such simplicity is rare, even for seemingly simple solid+solid mechanochemical transformations. For example, during the LAG mechanosynthesis of MOF‐74 from the binary solid mixture of ZnO and 2,5‐dihydroxyterephthalic acid, TRIS‐XRD analysis revealed impressive complexity (Figure 6 (I)).^[69]^ Within 20 minutes of ball milling, the reagents had already passed through three crystalline intermediate phases, before transforming into a fourth intermediate structure and finally yielding the MOF‐74 structure after 90 minutes of milling. Enormous intricacies were also captured by TRIS‐XRD during the mechanochemical cocrystallization of nicotinamide and anthranilic acid.^[70]^ Prior to reaching the final cocrystal product, ball milling caused numerous intermediate polymorphic transformations to occur.

It is, in principle, possible to solve the structure of intermediate phases directly from TRIS‐XRD data.^[11]^ Where this is not possible, as is often the case,^[71]^ TRIS provides the unique opportunity to both identify and “trap” short‐lived intermediates for subsequent ex situ analysis. In this way TRIS methods have proved vital for identifying new molecules and materials.[^72^, ^73^, ^74^] During the mechanochemical cocrystallization of pyrazinamide and malonic acid, TRIS‐XRD investigations revealed that a metastable cocrystal (form II) formed first, followed by its rapid conversion into the known thermodynamically stable phase (form I).^[75]^ The structure of form II could only be solved by stopping the reactor once TRIS data showed it to have formed. In this way, a sample of form II could be isolated and characterized using ex situ powder XRD data. Similarly, TRIS‐XRD was used to follow the ball‐milling process of zeolitic imidazolate framework 8 (ZIF‐8).^[76]^ During milling, the ZIF‐8 sample initially lost its crystallinity, before unexpectedly recrystallizing upon further milling. By using TRIS‐XRD, the authors found that recrystallization often yielded a previously unknown and short‐lived framework topology, which could be isolated for ex situ structure determination.

Growing evidence suggests that the bulk mechanisms of mechanochemical transformations can only be visible by using multiple TRIS techniques.^[52]^ During the mechanosynthesis of Cd(HO_3_PPh)_2_ from cadmium acetate dihydrate and phenylphosphonic acid,^[77]^ thermographic measurements exhibited clear endothermic events. Remarkably, subsequent TRIS‐XRD data revealed these events to correspond to the stepwise release of water from the hydrated starting phase, thereby confirming a stepwise mechanism of formation. In a similar study,^[77]^ the mechanochemical formation of a 1 : 1 oxalic acid and pyrazinamide cocrystal was also followed by TRIS thermometry. A discontinuity in the temperature profile matched well with the onset of the amorphization of the starting reagents, as observed by subsequent TRIS‐XRD analysis.

The complementarity of TRIS analytical methods has sparked interest in monitoring mechanochemical syntheses using multiple techniques simultaneously. During the mechanochemical synthesis of ZIF‐8,^[56]^ TRIS‐XRD suggested the direct conversion of the starting reagents (ZnO+2‐methylimidazole) into the final ZIF product. However, the tandem use of TRIS‐Raman spectroscopy revealed a more complex behavior. Although the basic ZIF‐8 structure was indeed formed within only minutes of ball milling, consistent with TRIS‐XRD analysis, the Raman frequencies showed molecular‐level distortions in the structure that persisted much longer into the milling process, before finally finding their place in the equilibrium structure (Figure 6 (II)). Recent developments in TRIS‐XRD analysis have since corroborated the Raman spectroscopy study,^[12]^ which suggests that more detailed TRIS methods may be required to complete the picture of the mechanochemical formation of ZIF‐8.

In a similar case, tandem TRIS‐XRD and TRIS‐Raman spectroscopy proved essential to reveal the mechanism of the mechanochemical formation of a 3‐dimensional Zn phosphonate material.^[78]^ During mechanosynthesis from a 1 : 1 mixture of reagents, TRIS‐XRD clearly showed the formation of a 3 : 2 intermediate phase. However, only through Raman spectroscopy could the reaction profile be completed, and the presence of the remaining starting material revealed.

*Physical Aspects*. In addition to the evolution of chemical species, the mechanisms of mechanochemical transformations depend on how the macroscopic structure of the material (mixing, state of aggregation, etc.) evolves (Figure 2). TRIS methods have also proved to be powerful in this regard. By following the resonant acoustic mixing (RAM) cocrystallization of carbamazepine and nicotinamide by TRIS‐XRD, the degree of bulk powder mixing was shown to greatly alter the outcome of a mechanochemical transformation.^[48]^ The role of bulk mixing in determining apparent mechanisms was also demonstrated for the ball‐milling synthesis of salts of glycine and oxalic acid.^[31]^ In this case, when particles of glycine were markedly smaller than those of oxalic acid, TRIS‐XRD revealed the preferential formation of the 2 : 1 (glycine : oxalic acid) phase. In contrast, the use of comparable sized particles favored the 1 : 1 product. Such studies highlight that—in contrast to solution reactions—it is the local (not global) composition that plays a dominating role in the evolution of the transformation.

Local/molecular mixing (i.e. comminution and formation of mobile intermediates; Figure 3), has also been successfully investigated by TRIS. For example, TRIS‐XRD has revealed that induction periods often comprise a significant degree of particle comminution.^[12]^ It has been suggested^[79]^ that this comminution is essential both as a mechanism to enhance molecular‐level mixing and to enhance the reactivity of the solid phases. In fact, high‐resolution TRIS‐XRD has indeed shown that significant microstructural distortions occur in powdered material during this period.^[12]^

Even beyond the mixing of the particles, TRIS has provided compelling evidence for dynamic molecular‐level mixing as a route to overcome the slow diffusion dynamics in solids. By studying the cocrystal formation between benzoic acid and 2‐pyridone, Lukin et al. demonstrated that ball milling drives molecular diffusion through the continuous comminution and growth of solid particles.^[80]^ After isotopically labeling the starting reagents, TRIS‐Raman spectroscopy was used to track vibrational shifts associated with deuterium exchange between compounds during milling. This TRIS study provided strong evidence for milling‐induced mass exchange. Further evidence was obtained through a combination of TRIS‐XRD and TRIS thermometry, which also showed that molecular rearrangements can seemingly occur in the solid state. Using as an example the base‐catalyzed benzil‐benzilic acid rearrangement,^[81]^ TRIS‐XRD analysis indicated that the rearrangement could occur under ball‐milling conditions without signs of bulk amorphization. Tandem thermographic measurements confirmed that the exothermic rearrangement occurred at the same time as observed in the diffraction pattern, strongly suggesting no underlying amorphous phase was present. However, while these data do suggest a solid‐state rearrangement, it remains unclear how the second solid (the base catalyst, KOH) is able to enter the crystalline lattice of the benzil species to allow the chemical reaction.

### 3.2 Macrokinetics

Kinetic analysis has been a powerful tool for indirectly probing mechanisms associated with chemical and physical processes. The development of TRIS analyses has reinvigorated this field of investigation in mechanochemistry, presumably because of the provision of more complete datasets for analysis. We note, however, that true kinetics (i.e. pertaining to elementary processes) continue to remain outside the scope of mechanochemical investigation. It is worth mentioning, however, that advanced analytical kinetic models are coming close to bridging this gap.^[82]^

Kinetic analysis of ball‐milling reactions has generally focused on two streams of research. In the first, macrokinetic analysis has focused on elucidating chemical aspects of the transformation. This includes studying the thermodynamic hierarchy of phases or feedback phenomena. In the second stream, kinetic analysis focuses on studying the bulk physical aspects of mechanochemical transformations, such as powder mixing, rheology, the influence of milling parameters (e.g. ball mass and size), and the nature of induction periods.

*Chemical Aspects*. Extensive efforts have been devoted to studying elementary stages of chemical transformations induced by single pulses of (or well‐defined) mechanical energy.[^83^, ^84^, ^85^, ^86^, ^87^, ^88^, ^89^, ^90^, ^91^, ^92^] However, similar detail is not yet possible for “real‐world” mechanochemical reactors, such as ball mills. Despite this, some recent studies have explored the use of TRIS technologies to push the boundaries of our molecular‐level understanding of bulk mechanochemical reactions through kinetic investigations.

Analysis of TRIS‐XRD ball‐milling kinetic curves led Lukin et al. to suggest autocatalytic nucleation and growth processes in the polymorphic transformation of a cocrystal of benzamide and nicotinamide.^[52]^ Autocatalytic behavior is being increasingly observed in mechanochemical transformations, as evidenced through TRIS analysis. During the ball‐milling synthesis of Ca[CO(NH_2_)_2_]_4_(H_2_PO_4_)_2_, water is released as a by‐product. When monitored by TRIS‐Raman spectroscopy, an autocatalytic kinetic model, dα/dt=kα(1-α) could be successfully fitted to the reaction profile. This strongly suggests that the released water causes self‐acceleration of the mechanochemical reaction. Similar autocatalytic behavior based on water production has also been observed ex situ.^[47]^

The extraction of thermodynamic landscapes has also been proposed based on the kinetic analysis of TRIS data. Following the kinetics of cocrystal formation as a function of jar temperature by TRIS‐Raman spectroscopy allowed an activation barrier for the transformation to be extracted.^[65]^ An apparent activation barrier to cocrystallization was found to be on the order of 15 kJ mol^−1^, comparable to the energy of the hydrogen‐bonding interactions that stabilize the reagent and product phases.

It has been shown by ex situ analysis that the starting form of a solid can influence the available mechanochemical transformations.^[93]^ In a striking example of TRIS‐Raman spectroscopy, this concept was further investigated using a simple Knoevenagel condensation reaction involving barbituric acid.^[94]^ By changing the origin of the barbituric acid (i.e. by using different barbituric acid cocrystals as starting reagents), the rate of the Knoevenagel condensation could be changed by over an order of magnitude. The authors suggest this to result from both the relative thermodynamic stability of the starting cocrystal phases and the nucleophilicity of the barbiturate. These early studies are very promising, and suggest much can be still learnt about mechanochemical energy landscapes through kinetic analysis.

*Physical Aspects*. Any chemical or physical transformation that occurs during mechanical treatment (e.g. ball milling) depends on the ability of the reactant powders to come into contact and the probability of the milling media contacting the powder. For this reason, kinetic analysis of physical phenomena has been of great interest for TRIS methods.

**Figure 7:**
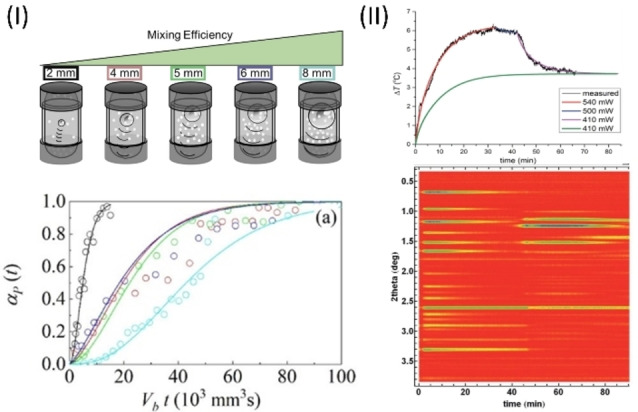
Kinetic analysis of TRIS data reveals new insights into the physical mechanisms of mechanochemical transformations. I) Ball‐milling synthesis of 1‐chloro‐3‐ethyl‐5,5′‐dimethylhydantoin as followed by TRIS‐XRD. The phase fraction $\alpha_{p}\left(t\right)$ is shown as a function of the ball volume ($V_{b}$ ) and time t . Figure adapted from Ref. [55]. Copyright 2021, American Chemical Society. II) Ball‐milling synthesis of ZIF‐8 monitored by tandem TRIS‐thermometry (top) and TRIS‐XRD (bottom). Kinetic heat flow models were fitted to thermometric data. Reproduced from Ref. [68] with permission from the Royal Society of Chemistry.

To better understand how ball–powder collisions affect the rates of mechanochemical transformations, TRIS‐XRD was used to follow a polymorphic transformation in caffeine.^[95]^ The original single‐phase system quickly became a binary phase (reagent+product). Kinetic analysis suggested that this conversion progressively reduces the number of ball–powder collisions that convert further reactant. This effect could be minimized by increasing the volume of the milling ball, thereby maximizing the probability of ball–reagent collisions at each impact. This finding was further supported by kinetic analysis of TRIS‐XRD data obtained for a multicomponent organic chemical mechanosynthesis, which was explored also with milling balls of different sizes.^[55]^ By normalizing the kinetic profiles to the volume of the milling ball, Martins et al. showed clearly that milling reaction kinetics are conserved (Figure 7(I)). It follows that the physical kinetics dominate reaction rates in a ball‐milling transformation; efforts to interpret such kinetics in purely chemical terms (i.e. as in solution) risk misinterpreting the results.

At the same time, kinetic analysis of TRIS‐XRD profiles has demonstrated that understanding material rheology is equally important for interpreting reaction kinetics. During the mechanochemical cocrystallization of glycine and oxalic acid, analysis of the raw TRIS‐XRD profiles revealed first order kinetics.^[31]^ However, by further considering the total scattering intensity, kinetic analysis instead revealed a first order “caking” of powder, and zeroth order chemical kinetics. In this way, TRIS‐XRD clearly demonstrated that significant rheological changes can occur during ball milling and have a significant impact on the observed transformation.^[47]^ Moreover, these rheological effects were suggested to be responsible for discontinuities in the kinetic profile of this same transformation.^[31]^

In addition to providing insights into powder and milling body behavior, TRIS‐XRD investigations have proved essential for understanding the general nature of reaction profiles (Figure 3). For example, induction periods have been reported with both loss of crystallinity^[96]^ and the absence of any notable changes in the X‐ray diffraction,^[81]^ likely because of the accumulation of defects.[^13^, ^24^, ^79^] Although it is not yet understood exactly what occurs during this poorly crystalline period, these induction periods have been suggested to result from requirements to mechanically activate the powder material prior to reaction.^[81]^ Evidence for such a mechanism has recently been obtained through new developments in TRIS‐XRD which allow for the accurate measurement of microstructure during ball milling, promising to shed light on this phase of a mechanochemical reaction.^[12]^

The structure of the powder is of course essential for determining the mixing and collision frequencies, and hence reaction kinetics.^[19]^ However, the evolution of temperature during ball milling can also be an important factor in determining the course of a mechanochemical transformation.[^97^, ^98^, ^99^, ^100^] Temperatures tend to plateau during ball milling as a result of heat dissipation through the jar.^[50]^ However, complex profiles indicate changes in the chemical or physical properties of the system. Užarević and co‐workers measured the thermal evolution of the reactor during the formation and amorphization of ZIF‐8 by ball milling.^[68]^ By using analytical heat flow kinetic models (Figure 7 (II)), the TRIS thermometric profiles were decomposed into unique reaction regimes. Each regime was found to be characterized by unique frictional properties, and hence heat flow kinetics, consistent with the sample undergoing various structural transformation. Such studies provide an intriguing view of how the bulk powder provides feedback to the local conditions (and hence apparent reaction kinetics) during ball milling.

### 3.3 Control of Mechanochemical Transformations

The study of mechanochemical mechanisms continues to reveal new—often unexpected—parameters that can be used to exert some degree of control over the transformation. It is becoming increasingly clear that in many cases we simply do not yet know what we do not know. Importantly, many control parameters are unique as compared to the better‐known field of solution chemistry. In view of this, obtaining control over mechanochemical transformations has proved to be a great—albeit exciting—challenge.

*Role of Different Initial Solid Forms*. Unlike in solution chemistry, the starting crystal forms seem to play an important role in dictating the outcome of a mechanochemical transformation.^[94]^ For example, the outcome of the mechanochemical transformation of chlorpropamide^[93]^ depends on which polymorphic form is taken as the initial reactant. The careful selection of the starting solid form is especially important in the case of hydrates and solvates. Only traces of cocrystal product are formed when anhydrous forms of l‐serine and oxalic acid are treated mechanochemically. In contrast, if either regent is present in its hydrated form, the milling transformation is found to occur quickly and quantitatively.^[101]^ It was, therefore, suggested that water in any form^[102]^ appears to facilitate the transformation. Further evidence for such a mechanism was obtained by TRIS‐XRD using the mechanochemical reaction between CdCl_2_.H_2_O and cyanoguanidine (cnge).^[66]^

*Control by Additives*. In addition to modifying the solid form of the starting solids, one can exert control over mechanochemical transformations by the intentional addition of an extra component to the mixture. In this way one can alter both the rates^[103]^ and mechanisms of the transformation. Such additives include seeds of solids, solvents, and polymers. The influence of small amounts of heterogeneous seeds was shown for the polymorphic transformation of benzamide form I in to form III, which can be initiated using small amounts of nicotinamide as seeds.^[104]^ ^15^N ssNMR experiments with doped and undoped reactants confirmed that the seed molecules are incorporated into the lattice of benzamide.^[104]^ TRIS‐XRD experiments of mixtures with different molecular ratios showed that the conversion process starts immediately after the grinding process is initiated.

Wilke et al. were able to elucidate the mechanism for the mechanochemical synthesis of two hybrid lead iodide compounds by TRIS XRD.^[105]^ By investigating different steps in the synthesis they could show that the formation of one of the compounds can be influenced by seeding, thus indicating that seeding could also be a beneficial approach for potential industrial applications.

Germann et al. investigated the polymer‐assisted mechanochemical cocrystallization of (caf):(glu) using in situ X‐ray powder diffraction.^[103]^ Their data reveal that the rate of cocrystallization is nearly doubled compared to the experiment under neat grinding conditions. Interestingly, in contrast to LAG experiments, the amount and weight of the added polymer does not affect the reaction rate, which indicates that small quantities of polymer are sufficient for a catalytic effect.

Many TRIS studies indicate that control over the polymorphic outcome of a milling experiment can be obtained through careful choice of the liquid additive in LAG reactions.[^50^, ^106^] In these examples, the polarity of the solvents used has a decisive influence, whereas in other cases the solvent can also act as a template.[^107^, ^108^] The templating effect of solvent molecules for the mechanochemical synthesis of covalent organic frameworks (COFs) was identified on the basis of TRIS‐XRD data.^[108]^ During the synthesis of different COFs, the 1,4‐dioxane used as the liquid additive was incorporated in the intermediate layered‐structured solvates. The 1,4‐dioxane molecules act as a template during the pore formation in the COF assembly, inhibiting initial π‐π stacking while promoting self‐assembly of 2D COF sheets.

*Control by the Mechanoreactor Conditions*. Growing evidence suggests that transformations conducted mechanochemically depend on more than just the nature of the chemical reagents added to the reactor.[^6^, ^109^] During the mechanochemical cocrystallization of nicotinamide and adipic acid, Germann et al.^[49]^ showed by using TRIS‐XRD that the reaction product changes depending on the choice of the jar or ball material as well as the number of milling balls used. By tuning the material of the milling apparatus, the authors isolated a new polymorphic form, thereby demonstrating a new route to access polymorphs without the need for further additives. The influence of the milling jar material has also been explored ex situ in various cases.[^110^, ^111^, ^112^]

It seems that one need not even change the reactor material to manipulate mechanochemical transformations. For a ternary cocrystal system, TRIS‐XRD data showed that reducing the milling frequency leads to a different mechanism involving the formation of binary phases in a first step prior to the formation of the ternary compound, whereas higher frequencies lead to a direct conversion.^[113]^ Although the same product was ultimately achieved, these early findings strongly suggest that product formation should be tunable through this same milling parameter.

In a similar fashion, a mechanochemical transformation can also be manipulated by varying the mass of the reagent powder. For the mechanochemical cocrystallization between theophylline and benzoic acid,^[114]^ TRIS‐XRD showed there was an immense dependence of the induction period and reaction times on the ball‐to‐powder mass ratios. The time required to complete the transformation was significantly shorter with a higher ball to reactant ratio. This presumably stems from more efficient mixing in smaller powder quantities, and the higher number (and energy) of collisions per particle as the powder volumes are scaled down. In another study using TRIS Raman spectroscopy, the reaction rates could be tuned by changing only the frequency and intensity of collisions.^[115]^ This was achieved by varying the ball mass, size, and quantity, while keeping the other reaction parameters constant.

Other reactor parameters, such as the bulk reaction temperature, have also been found to have a remarkable influence over mechanochemical transformations, akin to solution chemistry. Ball milling of CdCl_2_ and cyanoguanidine (cnge) was found to be sensitive to small changes in the bulk temperature.^[66]^ When milling under noncontrolled temperature conditions, the 1 : 2 mixture reacted to form the expected one‐dimensional coordination polymer, Cd(cnge)_2_Cl_2_, even when heating from the milling balls reached bulk temperatures of up to 32 °C. However, when the bulk temperature of the jar was increased to 50 °C, TRIS‐XRD revealed the formation of the 3‐dimensional coordination polymer, Cd(cnge)Cl_2_, as an intermediate in the formation of the final product with the 3‐dimensional structure. The rate of formation of the intermediate compound increased with temperature, from about 8 %/min at 50 °C to over 13 %/min at 70 °C.

Cindro et al. investigated different compounds and reported how the reaction temperature can influence the outcome of a mechanochemical reaction. A particular convincing example is a one‐pot reaction of acyl azide and diamine reactants to afford amide, amine, or urea products depending on the chosen temperature during milling.^[97]^

## 4 Prospects for TRIS Mechanochemistry

**Figure 8:**
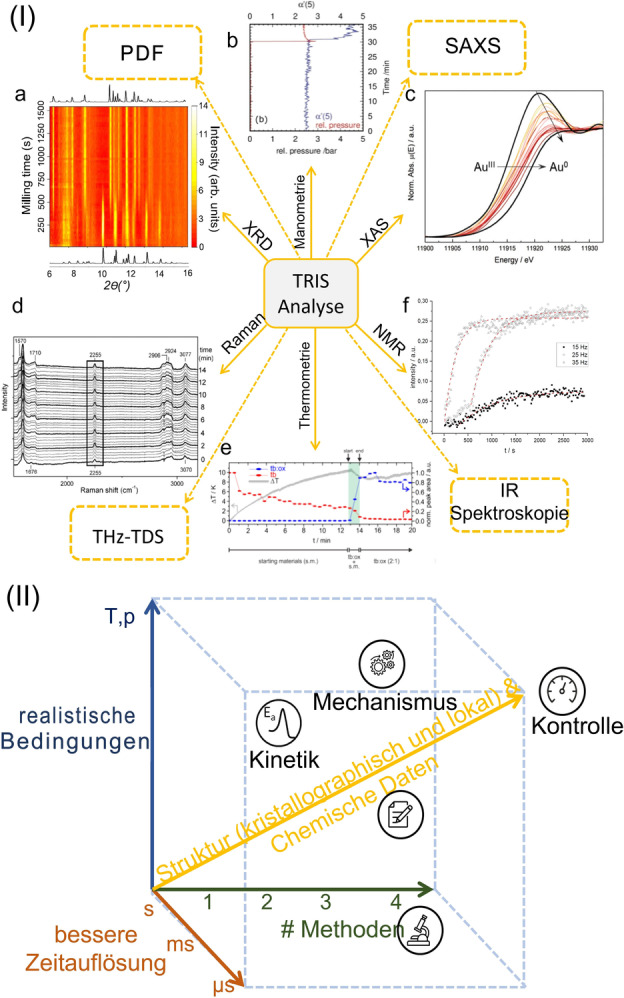
Established and potential methods (dashed lines) for time‐resolved in situ investigations of mechanochemical reactions. I) a) Typical XRD measurement for a reaction between bis(2‐nitrophenyl)‐ and bis(4‐chlorophenyl)disulfide,^[12]^ Copyright 2021, Nature Publishing Group. b) Relative pressure during the ball milling of Zn and S_8_,^[62]^ Copyright 2015, Wiley‐VCH. c) XANES data for the mechanochemical formation of gold nanoparticles. Figure adapted from Ref. [44] with permission from the Royal Society of Chemistry. d) Raman spectra of cocrystal formation from nicotinamide and suberic acid,^[39]^ Copyright 2014, Wiley‐VCH. e) Temperature development during the mechanochemical cocrystallization of theobromine and oxalic acid dihydrate. Figure adapted from Ref. [36] with permission from the Royal Society of Chemistry. f) ssNMR data showing product evolution as a function of time during the formation of zinc phenyl phosphonate.^[58]^ Copyright 2020, Elsevier. Dashed boxes: potential extensions of the range of TRIS methods: Terahertz time‐domain spectroscopy (THz‐TDS), small‐angle X‐ray scattering (SAXS), and pair distribution function (PDF) analyses. II) Schematic representation describing the pathway to more detailed chemical information from time‐resolved in situ (TRIS) studies.

Only within the last decade have methods for TRIS characterization of mechanochemical reactions become established. However, in this short time they have already contributed enormously to progressing our understanding of mechanochemical transformations (Figure 8 (I)). In particular, the ability to directly follow the evolution of crystallographic and local (molecular) structures in real time has opened new dimensions in research. By using TRIS methods, it is now possible to readily study reaction pathways and short‐lived metastable phases, map out energetic and thermodynamic aspects of milling reactions, and identify routes to selectively alter the outcome of a reaction. That said, we are only in the early days of TRIS studies, with exciting possibilities on the horizon.

It is becoming increasingly clear that no single analytical technique alone can provide the whole picture (Figure 8 (II)). This arises from the fact that mechanochemical reactions involve an interplay of physical and chemical phenomena over many orders of magnitude in temporal and spatial dimensions. The combination of two or more analytical methods offers the possibility to compensate for the respective weaknesses of individual methods.

Despite the availability of numerous TRIS methods (Figure 8 (I)), improved and new TRIS methods are still required to achieve characterization at the time and length scales necessary to fully capture mechanochemical reactions. For example, the development of pair distribution function (PDF) analysis will fill the gap between local environment characterization (XAS) and probing of the bulk crystalline state (XRD). Similarly, small‐angle X‐ray scattering (SAXS) will provide integral information about particle sizes. One can also expect applications of low‐frequency vibrational spectroscopy—such as terahertz time‐domain spectroscopy (THz‐TDS)—to be an important tool for characterizing crystal lattice structure, where Raman spectroscopy fails. With new mechanistic targets being proposed by theoreticians,[^116^, ^117^] we can expect a continual need to push experimental capabilities to their limits. Akin to developments in molecular sciences, as we continue to push the boundaries of our analytical capabilities, we will grow increasingly reliant on theoretical models.

When developing new methods for TRIS experiments one must balance the demand for a sample environment that is as close to reality as possible with the effort to obtain high‐quality data from the experiment (Figure 8 (II)). Against this background, the question arises as to whether the measured data are representative of mechanochemical reactions for different mill types or only depict a result that can be achieved under these experimental conditions. A striking example is the observation that reactions conducted in steel jars (often used in a laboratory) can differ from those performed in plastic jars (used for TRIS measurements).^[49]^ Moreover, most TRIS methods have so far only been developed for use on a single type of mechanochemical reactor—typically vibratory ball mills. It is, therefore, not yet possible to follow many academically and industrially relevant processes by TRIS. In this sense, there is much work to be done to develop TRIS for a wider range of reactor platforms, and to learn how to robustly transfer mechanochemical reactions between different reactor platforms.

In view of the far‐reaching applications of mechanochemistry as a “chemical innovation that will change our world”,^[5]^ interest is quickly growing in the use of TRIS methods. Alongside the needed continued developments, it will soon become necessary to establish accepted protocols[^40^, ^118^] to guide newcomers through the practical lessons learned over years of development. In this regard, it may prove useful to agree on standard test systems to benchmark new setups. As TRIS methods evolve, new techniques will certainly be added to the available range of methods. Along with improved sampling methods and data collection strategies, we will be able to obtain new information from time‐resolved in situ data. We have only scratched the surface for the applications of TRIS monitoring in mechanochemistry. There is no doubt that this field will continue to grow and equip us with the necessary knowledge to unleash the potential of mechanochemistry for the green design of materials.

## Conflict of interest

The authors declare no conflict of interest.

## Biographical Information

*Adam A. L. Michalchuk is a senior scientist in the Department of Materials Chemistry at the Federal institute for Materials Research and Testing (BAM) in Berlin. He obtained his PhD from the University of Edinburgh, UK, working in the fields of theoretical and solid‐state chemistry. His current research focuses on combining experiment and simulation to investigate the mechanical reactivity of solids*.

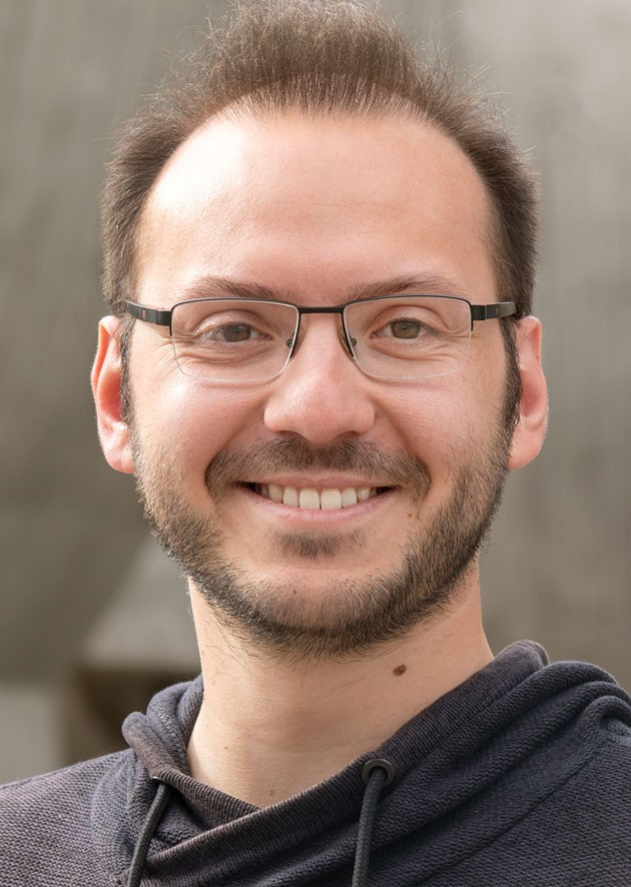

## Biographical Information

*Franziska Emmerling is head of the Department of Materials Chemistry at the Federal institute for Materials Research and Testing (BAM) in Berlin. With an educational background in inorganic chemistry, she obtained her PhD at the Department of Chemistry at the Albert‐Ludwigs University in Freiburg. Her fields of research include in situ investigations of crystallization processes, nanoparticle formation, and mechanochemistry*.